# Bending light via adiabatic optical transition in longitudinally modulated photonic lattices

**DOI:** 10.1038/srep15805

**Published:** 2015-10-29

**Authors:** Bin Han, Lei Xu, Yiling Dou, Jingjun Xu, Guoquan Zhang

**Affiliations:** 1 The MOE Key Laboratory of Weak Light Nonlinear Photonics, School of Physics and TEDA Applied Physics Institute, Nankai University, Tianjin 300457, China

## Abstract

Bending light in a controllable way is desired in various applications such as beam steering, navigating and cloaking. Different from the conventional way to bend light by refractive index gradient, transformation optics or special beams through wavefront design such as Airy beams and surface plasmons, we proposed a mechanism to bend light via resonant adiabatic optical transition between Floquet-Bloch (FB) modes from different FB bands in longitudinally modulated photonic lattices. The band structure of longitudinally modulated photonic lattices was calculated by employing the concept of quasi-energy based on the Floquet-Bloch theory, showing the existence of band discontinuities at specific resonant points which cannot be revealed by the coupled-mode theory. Interestingly, different FB bands can be seamlessly connected at these resonant points in longitudinally modulated photonic lattices driven by adiabatically varying the longitudinal modulation period along the propagation direction, which stimulates the adiabatic FB mode transition between different FB bands.

Photonic lattice has provided a versatile platform to manipulate the flow of light due to its designable geometric configuration therefore its band structure and optical properties, and various novel optical effects such as diffraction management^1^,^2^, lattice solitons^3^,^4^,^5^,^6^,^7^, and Anderson localization^8^,^9^ were reported. Recently, photonic lattices were engineered to visualize with optical waves a wide variety of quantum mechanical effects such as Bloch oscillations^10^,^11^,^12^,^13^, Zeno effect^14^,^15^, Zener tunneling^16^,^17^,^18^, and PT-symmetry^19^, to mention just a few. In addition, the introduction of longitudinal periodic modulation along the propagation direction of the lattices, which mimicks the dynamic action of external driving fields on quantum systems, may result in interesting optical-analogies of quantum coherent effects such as direct and indirect optical transition^20^,^21^ via spatial Rabi oscillation^22^ under the phase-matching condition, inhibition of light tunneling^23^,^24^ due to coherent destruction of tunneling^25^,^26^ in longitudinally modulated arrays with out-of-phase modulation between neighboring channels and subdiffractive propagation of light in bi-periodic arrays of fibers^27^ and photonic crystals^28^. More quantum-optical analogies such as coherent population transfer and dynamic localization were also demonstrated in periodically curved waveguide arrays^18^,^29^,^30^,^31^.

It is well known that the band structure of the photonic lattices is very important to understand the optical properties of the corresponding photonic lattices. Tremendous efforts have been put to calculate the band structures of various photonic lattices, and great progress has been made in the literature^2^,^6^,^7^. For longitudinally modulated photonic lattices, the perturbed coupled-mode theory^20^,^21^,^27^ may not be able to give accurate configuration of the band structure therefore the optical properties of the lattices, especially when the longitudinal modulation depth is comparable to or even larger than the transverse modulation depth. In fact, Floquet-Bloch theory is widely employed to calculate the band structure of complicated photonic structures such as photonic topological insulators^32^,^33^,^34^,^35^. In this article, we will calculate the band structure of longitudinally modulated photonic lattices based on the concept of quasi-energy^36^ and Floquet-Bloch (FB) formalism, which have also been employed to explain quantum coherent effects in atomic or molecular systems driven by a temporally periodic field, especially by an intensive laser field, leading to the discovery of many novel atom-field interaction phenomena that cannot be explained by the general perturbation theory^26^,^37^,^38^,^39^. Band discontinuity and adiabatic optical transition between different FB modes are predicted at specific resonant points, which can be used to manipulate the flow of light in photonic lattices with controllable bending light trajectory.

## Results

### Quasi-energy and Floquet-Bloch theory in longitudinally modulated photonic lattices

The evolution of a light beam with a slowly varying electric field amplitude *ψ*(*x*, *z*) propagating along the *z* direction of a longitudinally modulated photonic lattice can be described by^20^


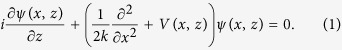


For simplicity but without loss of generality, the refractive index distribution of the photonic lattice is set to be *n* = *n*_0_ + *δn*cos(*ω*_*x*_*x*)cos(*ωz*), which is periodic in both *x* and *z* directions with *ω*_*x*_ and *ω* being the transverse and longitudinal angular frequencies of the refractive index modulation of the lattice, and the corresponding transverse and longitudinal lattice periods are *D* = 2*π*/*ω*_*x*_ and *T* = 2*π*/*ω*, respectively. *n*_0_ is the background refractive index and *δn* is the refractive index modulation depth. Therefore, the optical potential in Eq. (1) can be expressed as 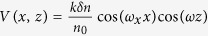, where *k* is the wave number of light in background. Note that the longitudinal refractive index modulation depth of the lattice is the same as that of the transverse one, therefore, it cannot be simply treated as a weak perturbation as done in the literature^20^,^21^,^27^. Such a strong longitudinal periodic modulation in the refractive index of the lattice, which is an optical analogy to a strong temporally periodic driving field in a quantum system^26^, is expected to have dramatic effects on the band structure of the lattice and therefore the beam evolution behaviors in the lattice. In addition, the longitudinal modulation period *T* is another important parameter that will have significant influence on the band structure of the lattice and also the dynamic propagation behavior of light in the lattice, as we will show in the following.

According to the Floquet-Bloch theory, the solution of Eq. (1) is of the form


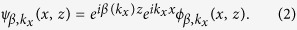


Where *β*(*k*_*x*_) is the propagation constant, which will be in the form of a series of bands *β*_*n*_(*k*_*x*_) with band index *n* = 1, 2, 3... for a photonic lattice^40^, *k*_*x*_ is the transverse wave vector. The FB modes 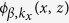 are periodic in both *x* and *z* directions, satisfying 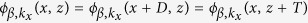. By substituting Eq. (2) into Eq. (1), one arrives at the stationary-like Schrödinger equation^36^


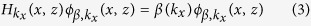


with


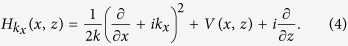


Here the eigen propagation constant *β*(*k*_*x*_), the analogy of quasi-energy in a quantum system driven by a temporally periodic field^26^,^36^, is real-valued and *z*-independent although the Hamiltonian 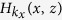 is *z*-dependent. One notices from Eq. (2) that modes 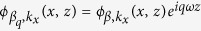, with *q* being an integer *q* = 0, ±1, ±2,..., are also solutions of Eq. (1) but with shifted eigenvalue *β*_*q*_(*k*_*x*_) = *β*(*k*_*x*_) − *qω*. Such shifted solutions are originated from the longitudinal periodicity of the optical potential *V*(*x*, *z*). Therefore, similar to the Brillouin zone due to the transverse periodicity of a photonic lattice, one can also restrict the eigenvalue bands *β*_*q*_(*k*_*x*_) into a unit region of a longitudinal reciprocal lattice vector *ω*.

To solve Eq. (3), one may expand the FB modes 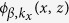 on the basis of a set of orthogonal basis


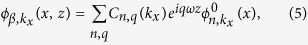


where *C*_*n*,*q*_(*k*_*x*_) is the expanding coefficients, 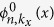 is the eigenstate on the *n*th-band of the unperturbed Eq. (3) (i.e., *V*(*x*, *z*) = 0) with the corresponding eigenvalue 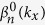, which is obviously in the form of a plane wave with a propagation constant 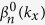 (for detailed concrete expressions of 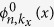 and 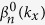, please refer to the corresponding text in Section **Methods**).

By substituting Eq. (5) into Eq. (3), one gets


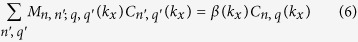


where 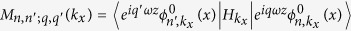 is the matrix element of Hamiltonian 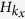, which, in the presence of an optical potential 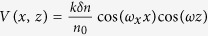, can be expressed as


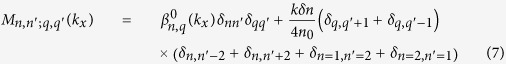


with 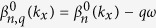. Detailed calculation of matrix element *M*_*n*,*n*′;*q*,*q*′_(*k*_*x*_) can be found in Section **Methods**. It is evident that the matrix is diagonal when the refractive index modulation vanishing (*δn* = 0), corresponding to a set of orthogonal plane waves with eigenvalues 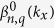, which form a series of shifted bands. Interestingly, different shifted bands indexed by (*n*, *q*) and (*n*′, *q*′) may intersect at specific resonant point 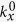 where 
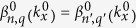
. According to the von-Neumann-Wigner degeneracy theorem^41^, by introducing a finite refractive index modulation *δn* which results in non-zero non-diagonal matrix elements in Eq. (7), such band crossing may be avoided when they belong to the same symmetry group of Hamiltonian 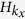, leading to band discontinuity at the resonant point 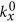.

### Band structure of longitudinally modulated photonic lattices

For a longitudinally modulated photonic lattice with an optical potential 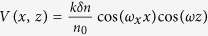, the non-diagonal matrix element in Eq. (7) is non-zero only when (*δ*_*q*,*q*′+1_ + *δ*_*q*,*q*′−1_)(*δ*_*n*,*n*′−2_ + *δ*_*n*,*n*′+2_ + *δ*_*n*=1,*n*′=2_ + *δ*_*n*=2,*n*′=1_) = 1 is satisfied. This means that only interactions among the nearest and the next-nearest bands have to be considered, which will dramatically simplify the band structure calculation. As an example while at the same time without loss of generality, let us consider a lattice with *D* = 10 *μ*m, *T* = 1300 *μ*m, *δn* = 5 × 10^−4^, and *n*_0_ = 2.3, which may be fabricated by using the proton-exchange or Ti in-diffusion techniques in lithium niobate crystals^19^,^42^,^43^ or the light-induction technique in photosensitive materials^28^. The wavelength of the incident light is set at *λ* = 0.6328 *μ*m. The band structure of the lattice can be obtained by directly solving Eq. (6).

**Figure 1:**
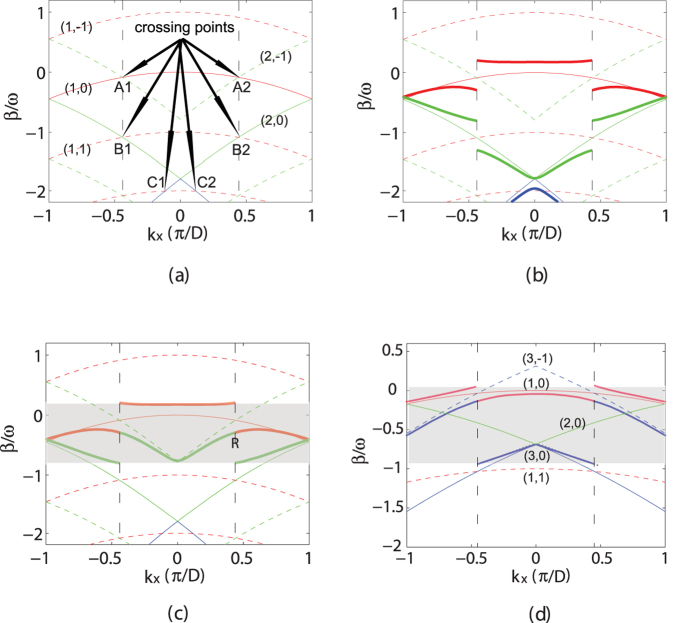
Band structures of longitudinally modulated periodic photonic lattices. (**a**) is the case with *V*(*x*, *z*) = 0, where the red, green and blue solid thin curves are the bands indexed by (*n*, *q*) = (1, 0), (2, 0) and (3, 0), respectively, while the colored dashed curves are the corresponding shifted bands. *A*1 and *A*2, *B*1 and *B*2, and *C*1 and *C*2 are crossing points where 
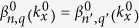
. The vertical dashed lines indicate the positions of the resonant transverse wave vector 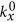. (**b**) is the longitudinally extended FB band structure and (**c**) is the corresponding reduced band structure of a lattice with *T* = 1300 *μ*m, and (**d**) is the reduced band structure of a lattice with *T* = 500 *μ*m, respectively, where the red, green and blue bold solid curves are the bands indexed by *n* = 1, 2 and 3, respectively. The point **R** in (**c**) is the resonant point at which the transverse velocity of FB modes in different bands is the same. The other lattice parameters are *D* = 10 *μ*m, *δn* = 5 × 10^−4^, and *n*_0_ = 2.3, which are the same for (**a**–**d**). The operating wavelength is set at 0.6328 *μ*m.

**Figure 2:**
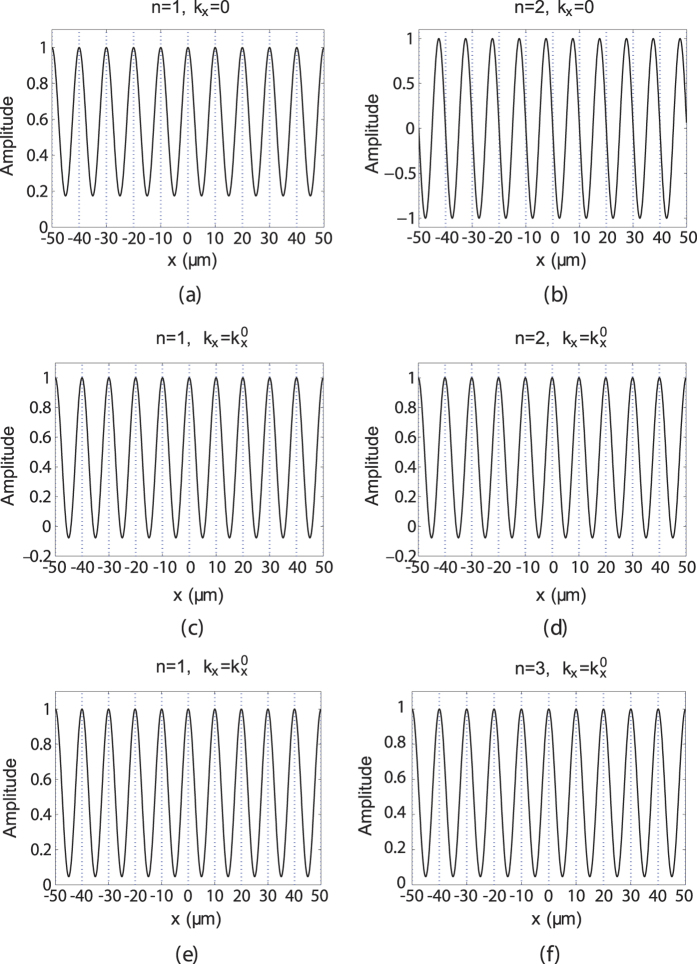
The field distribution of the FB modes with the transverse vector at *k*_*x*_ = 0 (a, b) and at the resonant points 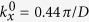 (c,d) and 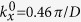 **(e,f)**, respectively. The longitudinal modulation period *T* is 1300 *μ*m for (**a**–**d**) and 500 *μ*m for (**e**,**f**), respectively, and the other lattice parameters are the same as those in Fig. 1. The vertical dashed blue lines denote position of the refractive index peak at the input lattice surface.

Figure 1(a) shows a portion of the band structure *β*(*k*_*x*_) for the case when *V*(*x*, *z*) = 0, where the red solid thin curve is the 1st-band with band index (*n*, *q*) = (1, 0) and the green and blue solid thin curves are the ones indexed by (2, 0) and (3, 0), while the dashed thin curves are the corresponding shifted bands, respectively. Note that, as a proof of principle and for clarity, other higher-order bands with band index *n* > 3 are not shown here. One notes that there are crossing points *A*1 and *A*2 between the two bands indexed by (1, 0) and (2, −1), and *B*1 and *B*2 between the two bands indexed by (2, 0) and (1, 1), respectively, at the resonant transverse wave vector 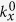 denoted by the vertical black dashed lines in Fig. 1. These crossings, according to the von-Neumann-Wigner degeneracy theorem^41^, will be avoided when a non-zero periodic refractive index modulation *δn*cos(*ω*_*x*_*x*)cos(*ωz*) is introduced, therefore, leading to band discontinuities at resonant points, as shown in Fig. 1(b). Note that the degeneracy is not lifted at other crossing points such as *C*1, *C*2 and those at the boundary of Brillouin zone because the crossing points belong to different symmetry groups of Hamiltonian 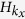 and they do not interact with each other^26^,^41^. Referring to the Brillouin zone in the transverse dimension, one could also restrict the extended FB bands into a reciprocal lattice primitive zone in the longitudinal dimension^36^,^44^, and the reduced band structure for the first two bands is shown by the bold red and green curves in the shadow region in Fig. 1(c). Here, higher-order bands with *n* ≥ 3 are not shown in the reduced shadow region for clarity. Typical FB modes with transverse wave vectors *k*_*x*_ = 0 and 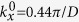 (at the resonant point) are shown in Fig. 2, which can be experimentally excited by two interfering plane waves or prism coupling method, or asymptotically implemented by simply launching a wide Gaussian beam with a well-defined central transverse wave vector matched with that of the FB mode^45^, as will be confirmed numerically in the following.

Figure 1(d) shows a portion of the reduced band structure of a lattice with a longitudinal lattice period *T* = 500 *μ*m, other lattice parameters are the same as those in Fig. 1(c). Again, only band indexed by *n* = 1 (band 1, the red bold solid curves) and band indexed by *n* = 3 (band 3, the blue bold solid curves) are shown in the reduced shadow region for clarity. In this case, crossing points between bands indexed by *n* = 1 and 3 are found to be avoided. Physically, such band discontinuity is originated from the introduction of periodic modulation on the refractive index in both the transverse and longitudinal dimensions. Note that the resonant point 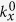, where the band discontinuity occurs, shifts when the longitudinal modulation period *T* changes. It can be easily confirmed that the band structure will return back exactly to the normal one with the band discontinuity located at the boundary of the first Brillouin zone (in this case, one calls it as band gap in general) when the longitudinal period *T* tends to be infinitely large. Therefore, the band structure of waveguide arrays without longitudinal modulation is only a special case in which the longitudinal modulation period *T* is infinitely large.

From the band structure in Fig. 1(c,d), one sees that, although there is band discontinuity in the same band, bands with different *n*-index will be smoothly connected at the resonant wave vector 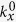. This is because the propagation constant, i.e., the quasi-energy in quantum mechanism, of the FB modes at the resonant point in both bands will be shifted by a same amount, as can be clearly seen in Eq. (7) where the off-diagonal matrix elements responsible for the band shift are exactly the same. One may also note that the field distribution of the FB modes at the resonant point, although in different bands, are the same (see Fig. 2(c–f)). Combining the fact that the resonant transverse wave vector 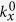 moves as the longitudinal lattice period changes, this provides a novel and efficient way to stimulate adiabatic optical transition between optical modes in different FB bands, which can be used to manipulate the flow of light in lattices, as we will discuss in the following section.

### Adiabatic optical transition and bending light in longitudinally modulated photonic lattices

**Figure 3:**
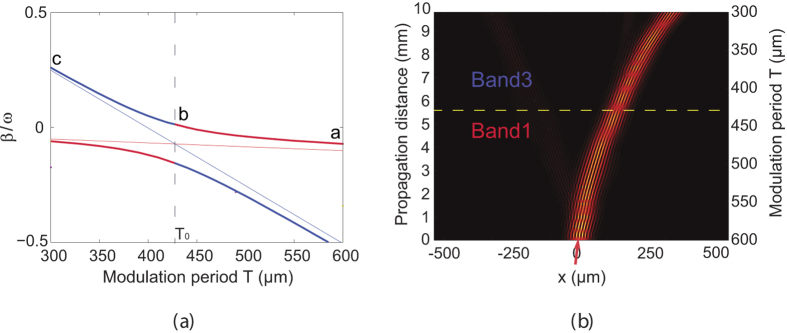
(**a**) Spectra of *β* as a function of longitudinal modulation period *T*. The FB modes in bands indexed by *n* = 1 and *n* = 3 are indicated by the red and blue bold solid curves, respectively. The thin straight lines show the spectra of *β* for corresponding lattices without longitudinal modulation. The dashed vertical line indicates the position of resonant longitudinal period *T*_0_ where adiabatic optical transition between FB modes from different bands occurs. (**b**) Beam propagation trajectory of a wide Gaussian beam in lattice with adiabatically decreasing longitudinal modulation period *T*. The yellow dashed line corresponds to the resonant longitudinal modulation period *T*_0_. The red arrow indicates the incident beam at *λ* = 0.6328 *μ*m with a transverse wave vector *k*_*x*_ = 0.7*π*/*D*. The other lattice parameters are *D* = 10 *μ*m, *δn* = 5 × 10^−4^ and *n*_0_ = 2.3, respectively.

Figure 3(a) shows a typical spectrum of propagation constant *β* as a function of longitudinal modulation period *T* at a specific incident transverse wave vector *k*_*x*_ = 0.7*π*/*D*. The lattice parameters are *D* = 10 *μ*m, *δn* = 5 × 10^−4^, and *n*_0_ = 2.3, respectively. One sees that the band indexed by *n* = 1 is smoothly connected to the band indexed by *n* = 3 at the resonant longitudinal period *T*_0_ = 428 *μ*m. Therefore, for a lattice with its longitudinal modulation period varying adiabatically from *T* = 600 *μ*m to *T* = 300 *μ*m (linearly here) along the propagation *z* direction, when one launches a FB mode with a transverse wave vector *k*_*x*_ = 0.7*π*/*D* into the lattice at the *T* = 600-*μ*m port (at point *a* in Fig. 3(a)), it will evolve along the upper spectral curve in Fig. 3(a) and transit adiabatically to the FB mode in band 3 at the resonant modulation period *T*_0_ (at point *b* in Fig. 3(a)). This is an exact optical analogy to spin exchange related to a rapid adiabatic crossing of resonance in nuclear magnetic resonance (NMR) when it is driven by a temporally varying magnetic field with adiabatically increased frequency^26^,^44^,^46^.

**Figure 4:**
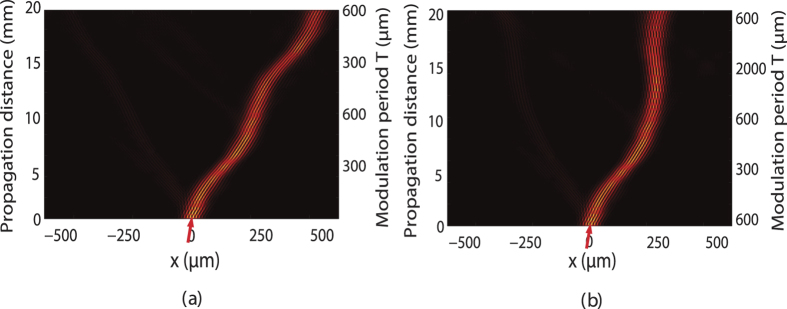
Snake-like light propagation in longitudinally modulated photonic lattices with adiabatically varying longitudinal modulation period *T*. (**a**) is the trace achieved by alternatively repeating the adiabatic FB mode evolution and optical transition from band 1 to band 3 and its reverse process shown in Fig. 3(a). (**b**) is the case by cascading adiabatic FB mode evolution and optical transition via a scheme band 1 → band 3 → band 1 → band 2 → band 1. The red arrow indicates the incident beam at *λ* = 0.6328 *μ*m with a transverse wave vector *k*_*x*_ = 0.7*π*/*D*. The other lattice parameters are *D* = 10 *μ*m, *δn* = 5 × 10^−4^ and *n*_0_ = 2.3, respectively.

Interestingly, such adiabatic mode evolution and optical transition can be used to control the beam propagation trajectory in the lattice because different FB modes are of different transverse propagation velocity determined by ∂*β*/∂*k*_*x*_. Figure 3(b) shows the beam propagation trajectory of a wide input Gaussian beam in the lattice with the same lattice parameters as those in Fig. 3(a) when the light evolves along the upper spectral curve (*a* → *b* → *c*) in Fig. 3(a). Here, a wide input Gaussian beam with a waist width being 5*D* is used in order to define a narrow spatial spectral content of the excitation. The central incident transverse wave vector of the light at the *T* = 600-*μm* input port is set at *k*_*x*_ = 0.7*π*/*D*. It can be easily confirmed numerically that over 80% of the input energy of the Gaussian beam will be converted into the target FB mode of lattice with its transverse wave vector *k*_*x*_ = 0.7*π*/*D*. One sees that, along with the adiabatic mode evolution and optical transition, the beam trajectory bends during its propagation in lattice. It is worth mentioning that the adiabatic optical transition is reversible and the FB modes in band 3 can transit adiabatically to the FB modes in band 1 via the reversed spectral curve (*c* → *b* → *a*) in Fig. 3(a). This means that, by alternatively repeating the adiabatic optical transition from band 1 to band 3 and its reverse process along the propagation *z* direction, light can be designed to propagate in the lattice with a snake-like trajectory, as shown in Fig. 4(a). More flexibly, different adiabatic mode evolution and optical transition processes may be combined and followed one by another one, and therefore complicated light propagation trajectory could be designed in principle. Figure 4(b) shows such an example, where a cascading adiabatic optical transition scheme (band 1 → band 3 → band 1 → band 2 → band 1) is designed, and a bending light with a designable large curvature trajectory is achieved. It is evident that any perturbation on the lattice parameter will influence the band structure of lattice, therefore the beam propagation trajectory. However, the practical tolerance on the deviation of lattice parameter is indeed very complicated and dependent on the requirement of specific applications, which is evidently deviated from the main topic of this paper and will not be discussed in detail here. Also, we suppose that the lattice is large enough so that the boundary effect is negligible.

## Discussion

Bending the light propagation trajectory in a designable way is desired in many practical applications such as beam steering and switching, beam navigation and even cloaking, and it may be achieved by designing the refractive index gradient along the light propagation trajectory^47^ or through transformation optics^48^,^49^ or by employing special beams with special wavefront structure such as Airy beams^50^,^51^,^52^ or Airy plasmons^53^,^54^,^55^,^56^,^57^,^58^. Note that the effective average refractive index of our longitudinally modulated photonic lattice is a constant *n*_0_, and the mechanism to bend the light propagation trajectory in our case relies on the adiabatic optical mode evolution and optical transition of FB modes among different FB bands, which is completely different from those through refractive index gradient or wavefront design mentioned above.

One may note that curved beam trajectory can also be observed in other transversely or longitudinally modulated lattices, for example, through Bloch oscillation in waveguide arrays with equally spaced increasing propagation constant in the transverse dimension^10^,^11^,^12^,^13^ and Rabi oscillation in waveguide arrays with weak homogeneous modulation in the longitudinal dimension^20^,^21^. However, the mechanisms leading to curved beam trajectories in Bloch oscillation and Rabi oscillation are totally different from that in our case. Bloch oscillations, which manifests themselves as transverse oscillations of the propagating light beam, is due to the excitation and beating of FB modes with equally spaced propagation constants^12^. For Rabi oscillation, the beam oscillates between two different FB modes from different bands, either directly or indirectly, depending on the momentum compensation of the transverse wave vector^20^. In our case, the longitudinal period of the photonic lattices changes linearly and adiabatically along the propagation direction, and the beam evolves continuously and adiabatically during its propagation, which experiences many FB modes in sequence in one band and then transits adiabatically into the FB modes in other band at the resonant point and evolves continuously and adiabatically again in the second band. Note that these phenomena are optical analogies of different quantum effects, i.e., Bloch oscillation, Rabi oscillation, and adiabatic electron spin exchange in NMR driven by a temporally varying magnetic field with adiabatically increased or decreased frequency, respectively. The mechanism in our case is also different from that based on the periodic longitudinal modulation on the coupling coefficient between neighboring waveguides through curved waveguide arrays^59^.

We would emphasize that the adiabatic optical transition shown in Fig. 3 is different from that achieved through Rabi oscillation reported in refs 20,21. Although both of them are driven by the longitudinal periodic modulation along the propagation *z* direction, the period of longitudinal modulation varies linearly and adiabatically along the propagation *z* direction in our case while it is kept to be the same in the Rabi oscillation case. In addition, two specific FB modes in different bands with different field distribution are involved in the Rabi oscillation, and the band structure of the lattice is invariant along the propagation dimension. While for our case, adiabatic mode transition occurs across the resonance point via band structure evolution and the two FB modes at the resonant point in two bands are of the same field distribution, as shown in Fig. 2(c–f). Note that the adiabatic optical transition is achieved by adiabatically scanning the longitudinal modulation period and the two involved FB modes are of the same field distribution, therefore it is always perfect with a 100%-conversion efficiency in our case. In contrast, the optical transition via Rabi oscillation is based on the parametric mixing between the FB modes from different bands, and the conversion efficiency may be relatively low, depending on the interacting bands^20^,^21^.

**Figure 5:**
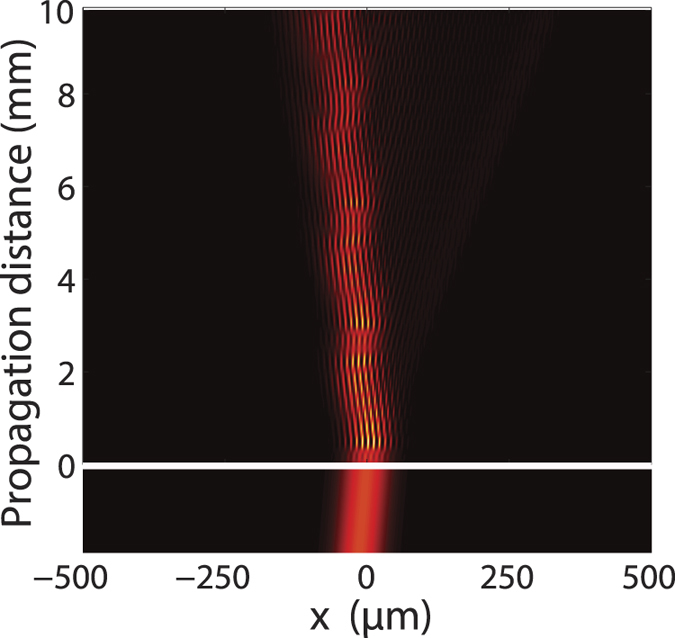
Effective negative refraction at the resonant transition point 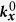 at the entrance surface (indicated by the white horizontal line) for a light beam incident from the homogeneous media with a refractive index *n*_0_ to the longitudinally modulated photonic lattice with *T* = 1300 *μ*m. The incident wavelength is set at 0.6328 *μ*m, and the lattice parameters are the same as those in Fig. 1(c).

The resonant transition point is specially interesting (see the point R marked in Fig. 1(c)). Such resonant transition points have been extensively studied in quantum system driven by a temporally periodic field, many novel effects such as driven quantum tunneling and coherent destruction of tunneling have been observed^26^,^36^,^41^. For the longitudinally modulated photonic lattices here, besides the adiabatic optical transition between FB modes in different bands, one can also achieve effective negative refraction near this special resonant transition point because of the smooth connection between different FB bands at the resonant point. From the reduced band structure shown in Fig. 1(c), one sees that the FB modes of band 1 and band 2 in Fig. 1(c) with a resonant transverse wavevector 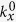 propagate in the same direction in the longitudinally modulated photonic lattice, leading to an effective negative refraction phenomenon, as shown in Fig. 5. The seemly fringe pattern along the beam propagation path in the modulated lattice is a discrete characteristic of the lattice, which occurs in all transversely modulated lattices^2^,^6^,^40^. Note that the observed negative refraction effect is determined by the band structure of the longitudinally modulated lattice, therefore, it occurs when the transverse wave vector *k*_*x*_ of the exciting beam is matched, no matter the longitudinal modulation at the interface is switched on smoothly or abruptly. It can be numerically confirmed that a slight deviation of the incidence beam from the resonant point within Δ*k*_*x*_ < 0.1*π*/*D* is acceptable for the observation of negative refraction. This is very different from that in a photonic lattice without longitudinal modulation, where the excited two FB modes in two different bands will propagate in different directions^1^,^40^,^60^.

In conclusion, based on the concept of quasi-energy and the Floquet-Bloch theory, we have succeeded in getting the accurate band structures of longitudinally modulated photonic lattices in which the perturbation approximation of the coupled-mode theory may not be applicable. Band discontinuity is observed due to the avoiding crossing effect at the resonant transverse wave vectors, where, on the other hand, different FB bands happened to be smoothly connected in the reduced band diagram, leading to interesting effects such as negative refraction at these resonant points. More interestingly, by adiabatically varying the longitudinal modulation period along the propagation direction, adiabatic optical transition between FB modes from different bands can be achieved with a perfect conversion efficiency, which can be used to bend the light propagation trajectory in the lattices, and designable snake-like beam propagation is achieved. This may have potential applications in beam steering, navigating and even cloaking.

## Methods

### Orthogonal basis 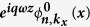


The state 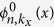 in Eq. (5) is the eigenstate of the unperturbed Eq. (3) (i.e., *V*(*x*, *z*) = 0) with the corresponding eigenvalue 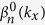. In order to compare with the band structure of lattices without longitudinal refractive index modulation, we employ the conventional band index scheme with positive index number (*n* = 1, 2, 3,...) in the reduced band-gap diagram for photonic lattices^40^,^61^, and the eigenstate is in the form


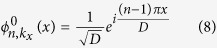


with 
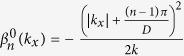
 when *n* = 1, 3, 5... is an odd integer, and


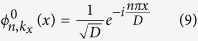


with 
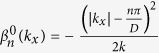
 when *n* = 2, 4, 6... is an even integer.

Let 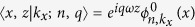. It is evident that the orthogonal basis satisfies^36^


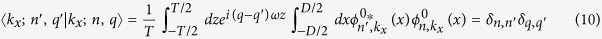


### Calculation of Matrix Element *M*_*n*,*n*′;*q*,*q*′_

The matrix element *M*_*n*,*n*′;*q*,*q*′_ of the Hamiltonian 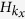 can be expressed as


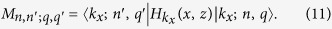


By taking the orthonormality of the eigenstate 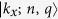 into consideration and with an optical potential 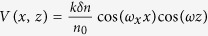, one arrives at


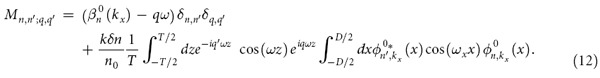


Here


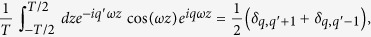


and


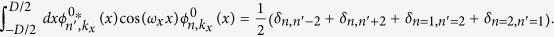


Therefore, one can get the matrix element


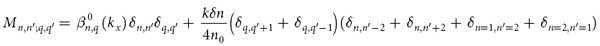


with 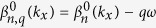.

### BPM

The beam propagation trajectories in the lattices shown in Figs 3(b),4 and 5 are simulated by employing the beam propagation method (BPM).

## Additional Information

**How to cite this article**: Han, B. *et al.* Bending light via adiabatic optical transition in longitudinally modulated photonic lattices. *Sci. Rep.* **5**, 15805; doi: 10.1038/srep15805 (2015).
